# Nail-patella-like renal disease masquerading as Fabry disease on kidney biopsy: a case report

**DOI:** 10.1186/s12882-020-02012-3

**Published:** 2020-08-13

**Authors:** Filippo Pinto e Vairo, Pavel N. Pichurin, Fernando C. Fervenza, Samih H. Nasr, Kevin Mills, Christopher T. Schmitz, Eric W. Klee, Sandra M. Herrmann

**Affiliations:** 1 Center for Individualized Medicine, Mayo Clinic, Rochester, MN USA; 2 Department of Clinical Genomics, Mayo Clinic, Rochester, MN USA; 3 Division of Nephrology and Hypertension, Department of Medicine, Mayo Clinic, 200 First St SW, Rochester, MN 55905 USA; 4 Department of Laboratory Medicine and Pathology, Mayo Clinic, Rochester, MN USA; 5 UCL Institute of Child Health, Great Ormond Street Hospital, London, UK; 6 Department of Biochemistry and Molecular Biology, Mayo Clinic, Rochester, MN USA; 7 Department of Health Sciences Research, Mayo Clinic, Rochester, MN USA

**Keywords:** Fabry disease, *LMX1B*, Nail-patella-like renal disease, Individualized medicine

## Abstract

**Background:**

Genetic changes in the LIM homeobox transcription factor 1 beta (*LMX1B*) have been associated with focal segmental glomerulosclerosis (FSGS) without the extra-renal or ultrastructural manifestations of Nail-patella syndrome (NPS) known as Nail-patella-like renal disease (NPLRD). Fabry disease (FD) is an X-linked lysosomal disease caused by the deficiency of alpha-galactosidase A. The classic form of the disease is characterized by acroparesthesia, angiokeratomas, cornea verticillata, hypertrophic cardiomyopathy, strokes, and chronic kidney disease. Podocyte myelin bodies on ultrastructural examination of kidney tissue are very characteristic of FD; however some medications and other conditions may mimic this finding.

**Case presentation:**

Here, we report on a female patient with chronic kidney disease (CKD), positive family history for kidney disease and kidney biopsy showing a FSGS lesion and presence of focal myelin figures within podocytes concerning for FD. However, genetic testing for FD was negative. After comprehensive clinical, biochemical, and genetic evaluation, including whole exome and RNA sequencing, she was ultimately diagnosed with NPLRD.

**Conclusions:**

This case illustrates the difficulties of diagnosing atypical forms of rare Mendelian kidney diseases and the role of a multidisciplinary team in an individualized medicine clinic setting in combination with state-of-the-art sequencing technologies to reach a definitive diagnosis.

## Background

Nail-patella syndrome (NPS; OMIM #161200) is an autosomal dominant disease caused by variants in *LMX1B* which encodes for the LIM homeobox transcription factor 1 beta that plays a critical role in the development of the limb structures, glomerular basement membrane in the eye, kidney, and neurons [1]. The classic phenotype of NPS involves nail dysplasia, reduction in flexion of the interphalangeal joints, elbows, and knees, patella abnormalities such as absence, hypoplastic or dislocation, and the presence of iliac horns on pelvis x-rays [2]. The disease is completely penetrant, however it has variable expressivity with inter- and intrafamilial variability. Renal involvement occurs in 30–50% of the individuals with NPS and end-stage renal disease (ESRD) is seen in 5% of the cases. Usually, the first sign of renal function impairment is proteinuria with or without hematuria [3]. However some individuals may present with renal involvement without skeletal or extrarenal manifestations, a condition known as Nail-patella–like renal disease (NPLRD) or *LMX1B*-associated nephropathy (OMIM #256020) [4].

Fabry disease (FD; OMIM #301500) is an X-linked inborn error of metabolism caused by pathogenic variants in *GLA* which encodes for the lysosomal enzyme alpha-galactosidase A (α-GAL). This enzymatic defect leads to the buildup of globotriaosylceramide (Gb3) and its derivatives such as globotriaosylsphingosine (lyso-Gb3) in different cell types, including endothelial cells, skin, podocytes, cardiomyocytes, and nervous system. The progressive accumulation of Gb3 causes the clinical features of FD that include angiokeratomas, cornea verticillata, gastrointestinal symptoms, proteinuria, progressive kidney disease, hypertrophic cardiomyopathy, and strokes [5]. Atypical variants of FD in which individuals present with only cardiac disease [6] or kidney disease are also recognized [7, 8]. Some genetic variants are specific for the classic or atypical forms. However, clear genotype-phenotype correlation does not exist [9]. Moreover, this is especially true in women who may be asymptomatic, oligo symptomatic, or present the classic form of the disease due to skewed X inactivation [10]. In men, the diagnosis of FD can be established with the identification of deficient α-GAL activity in leukocytes, plasma, or cultured cells.

However, in women, the enzymatic activity is an unreliable assay, so genetic analysis of *GLA* should be performed. Lyso-Gb3 measurement in plasma and urine may help clarify variants of uncertain significance identified by genetic testing, particularly in individuals with late-onset or atypical forms of the disease [11].

Herein, we report on a female proband with longstanding history of CKD, family history of CKD and a kidney biopsy showing a focal segmental glomerulosclerosis (FSGS) lesion on light microscopy and myeloid bodies in the podocyte on electron microscopy EM) examination, who was referred to our Institution with a suspicion of FD but who ultimately was diagnosed with NPLRD after exome and RNA sequencing in the kidney tissue.

## Case presentation

A 65 year-old white female patient had been previously evaluated an outside facility for recent development of edema and CKD. She had a history of hypertension diagnosed at age 58 on treatment with angiotensin II receptor blocker. At time of her initial evaluation (at an outside facility) her serum creatinine was 1.1–1.2 mg/dL and urine analysis demonstrated proteinuria +++. She underwent a kidney biopsy that showed a FSGS lesion and presence of focal myelin figures within podocytes (zebra bodies). *GLA* sequencing for FD was negative. She had no history of prior exposure to silica, amiodarone or hydroxychloroquine, which can be associated with lesions resembling zebra bodies on EM. Her mother was diagnosed with FSGS at age 69 years, then developed end-stage renal disease, 2 years later, and was on dialysis until her 80s when she died. The patient’s father had died due to liver cancer. She has two healthy brothers, ages 56 and 63, and two healthy sons who are 39 and 40 years old.

**Fig. 1:**
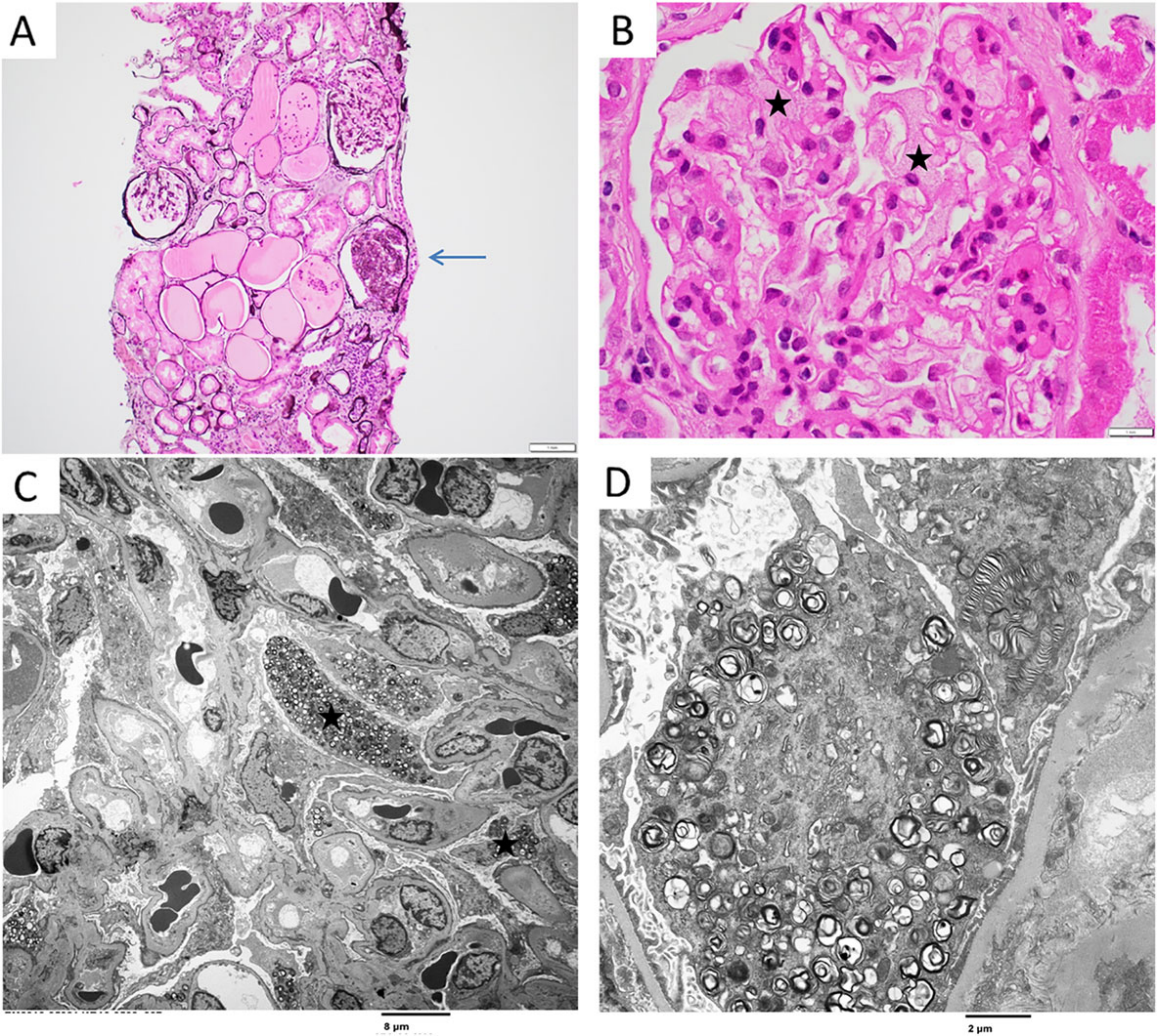
Kidney biopsy findings. **a** Low power image showing a glomerulus with segmental sclerosis (arrow) and focal tubular atrophy, interstitial fibrosis, and chronic inflammation (bottom of figure) (silver stain). **b** Some podocytes in this glomerulus (stars) show bubbly cytoplasm on H&E. **c** Low power electron microscopy image showing lipid inclusions (myeloid bodies) in some podocytes (stars) but not in the other cell types. **d** High power electron microscopy figure showing abundant myeloid figures within the cytoplasm of a podocyte

Due to the inconsistency between the EM findings and the genetic analysis, and to obtain further clarification regarding the diagnosis, the patient was referred to Mayo Clinic 4 years after the first evaluation. She had no history of acroparesthesias, angiokeratomas, transient ischemic attacks, hypohidrosis, parapelvic cysts or any other FD-related signs or symptoms [12]. One son had negative biochemical testing and the other had negative genetic testing for FD. Physical examination was unremarkable including no evidence of corneal abnormalities in ophthalmological evaluation. Laboratory evaluation is presented on Table 1. Cardiac and brain magnetic resonance imaging were unrevealing. To further evaluate the cause of her renal disease, she underwent a repeat kidney biopsy. On light microscopy (LM) there were 30% globally sclerotic glomeruli and one glomerulus showed segmental sclerosis with podocyte capping. Few glomeruli showed duplication of the glomerular basement membranes. There was mild tubular atrophy and interstitial fibrosis (Fig. 1a) involving 25% of the cortex sampled accompanied by mild chronic interstitial inflammation. Some podocytes with bubbly cytoplasm were seen on LM (Fig. 1b). No glomeruli were seen in the immunofluorescence. There was 3+ focal tubular casts staining for IgA, kappa, and lambda with 2+ IgM. The medullary tissue was negative for IgG, C1q, C3, albumin, and fibrinogen. On EM, several podocytes contained myeline figures resembling zebra bodies (Fig. 1c and d) with podocytes displaying mild to moderate foot process effacement involving approximately 30% of the total peripheral capillary surface area. No myelin figures were seen within endothelial cells, mesangial cells, peritubular capillaries, or tubular cells. No beaded collagen fibrils were seen within the glomerular basement membranes. *GLA* deletion/duplication testing for FD gene was repeated and was negative. Due to the inconclusive biochemical and genetic testing, she was referred to the Department of Clinical Genomics for further evaluation. To further evaluate the cause of her renal biopsy findings, RNA sequencing of kidney tissue was performed through a research program in the Center for Individualized Medicine [13]. No variants, aberrant expression, aberrant splicing changes, or allelic imbalance was detected within *GLA* by RNA analysis*.* Moreover, there was no sign of skewed X chromosome expression. To search for other possible genetic explanation for her symptoms, whole exome sequencing was performed on the kidney tissue and revealed a pathogenic variant in *LMX1B* (NM_002316.3:c.737G > A, p.Arg246Gln), which is associated with NPLRD in multiple families. The variant was Sanger confirmed to be germline on DNA extracted from the patient’s blood. Her renal disease progressed and 2 years after presentation to the Mayo Clinic the patient received a kidney transplant from a deceased donor.

**Table 1:** Biochemical evaluation

General blood test	Result	Normal Range
Hemoglobin (g/dL)	12	12–15.5
Leukocytes ×10(9)/L	6.6	3.5–10.5
Platelet ×10(9)/L	352	150–450
Sodium (mmol/L)	143	135–145
Potassium (mmol/L)	5.4	3.6–5.2
Bicarbonate (mmol/L)	25	22–29
Chloride (mmol/L)	105	98–107
Creatinine (mg/dL)	1.9	06–1.1
Blood urea nitrogen (mg/dL)	41	6–21
Albumin (g/dL)	3.6	3.5–5
Calcium (mg/dL)	9.1	8.9–10.1
Glucose (mg/dL)	98	70–100
Phosphorus (mg/dL)	4.7	2.5–4.5
C-reactive protein	< 3.0	< 8.0
PTH (pg/mL)	79	15–65
Total cholesterol (mg/dL)	361	< 200
Triglycerides (mg/dL)	283	< 150
Hepatitis B and C	Negative	Negative
Monoclonal protein	Negative	Negative
**Fabry disease studies**	**Result**	**Normal Range**
Alpha-Galactosidase, Leukocytes (nmol/h/mg)	35.3	> 23.1
Alpha-Galactosidase, Plasma (U/L)	0.17	0.074–0.457
Ceramide Trihex and Sulfatide, Urine	not detectable	not detectable
Gb3, Plasma (ug/mL)	6.5	< 7.5
Gb3, Urine (mg/mmol/Cr)	0.031	< 0.03
Lyso-Gb3, Plasma (ng/mL)	0.4	< 1.8
Lyso-Gb3, Urine (ng/mmol/Cr)	6.8	not detectable
**Urine studies**	**Result**	**Normal Range**
Hemoglobin, qualitative	Small	negative
Red blood cells	< 3	< 3 /HPF
White blood cells	4–10	1–10 < HPF
Casts, hyaline/LPF	occasional	not detectable
Fatty casts/LPF	occasional	not detectable
24 h proteinuria (mg)	6162	< 167

## Discussion and conclusions

Our case highlights the difficulties of diagnosing atypical forms of rare Mendelian kidney diseases, especially when kidney biopsy findings are suggestive of a genetic disease but diagnosis cannot be confirmed by routine evaluation. The diagnosis of NPS is associated with variable phenotypes, including nail changes present in 98% of the probands with the typical form of disease. The prevalence of NPS is estimated at one in 50,000 individuals, but likely underestimated due to the variable phenotypic severity in affected individuals. Genetic analysis of *LMX1B* can be informative when a clinical diagnosis is uncertain or for individuals with atypical forms of NPS. There is no clear genotype-phenotype correlation; however, our patient’s *LMX1B* pathogenic variant has been identified in other families presenting with FSGS without the extra-renal or ultrastructural manifestations of NPS similar to our patient’s phenotype [1]. Knockout mice studies have shown that reduced levels of the LMX1B protein causes NPS [14]. The Arg246 residue is located in the homeodomain of the LMX1B, is well conserved throughout evolution, and has been shown to have a critical role on *LMX1B* function. The Arg to Gln change reduces transcriptional activity causing a decrease of LMX1B level not sufficient to affect the nails and limbs [15]. Interestingly, patients diagnosed with typical NPS and with variants in the homeodomain are more likely to have kidney disease than patients with variants in other protein domains [16]. Moreover, the renal prognosis of patients with a p.Arg246Gln is worse than that of typical NPS nephropathy [17]. The renal biopsy of individuals with NPLRD are heterogeneous and may show minimal-change disease, normal EM, effacement of podocyte foot processes, or a FSGS lesion. However, the typical finding for NPLRD is the presence of deposition of type III collagen fibrils [15]. On the other hand, FD has very specific ultrastructural findings in the kidney biopsy. LM shows vacuolization of podocytes and distal tubular epithelial cells [18]. On EM, deposits of Gb3 appear as lamellated structures, called myeloid or zebra bodies. Still, these findings are not pathognomonic of FD since lamellar inclusions have been described in silicosis, and with the use of chloroquine, hydroxychloroquine, imipramine, and gentamicin [19]. Of note, there are anecdotal reports of FD phenocopy in individuals with myeloid bodies in the kidney biopsy that have negative evaluation for FD and unknown cause for the lipids deposition [20, 21].

The kidney findings and slightly elevated urinary lyso-Gb3 in our patient were concerning for FD, and led us to carry out RNA and DNA testing in search of a germline variant, somatic variant, or copy number change in *GLA,* but results were negative. Interestingly, there is no non-FD individual with increased urinary levels of lyso-Gb3 reported to date. Individuals with other causes for CKD might present with increased levels of urinary Gb3 but they have normal levels of lyso-Gb3 [22]. However, it is still not clear if individuals with other causes for renal phospholipidosis could also excrete lyso-Gb3. It is important to point out that in FD, myeloid bodies are usually present not only in the podocytes, but also in mesangial cells, endothelial cells, distal tubule, and interstitium whereas in our patient there were inclusions only in the podocytes similar to non-FD causes, and provides a clue that we were dialing with a disease other than FD [23]. The FSGS lesion in our patient is consistent with what has been reported in association with the identified *LMX1B* variant. Noteworthy, two additional families with our patient’s variant in *LMX1B* and myeloid bodies on the kidney biopsy were recently reported but no information on biomarker level was provided [24]. Thus, at this time it is not possible to establish a definite association between *LMX1B,* myeloid bodies and lyso-GB3 excretion, or if the deposition is secondary to lysosomal enzymatic deficiencies due to medications or infections. Together, these cases indicate that LMX1B-related diseases should be considered in the differential diagnosis of patient with renal biopsy showing myeloid bodies on EM.

It is important to highlight that obtaining definitive diagnosis to patients with inconclusive causes of CKD, such as the case reported, may prevent use of unnecessary therapies as this patient would not improve with enzyme replacement therapy with recombinant α-GAL which would have been initiated had the patient been misdiagnosed as FD. A multidisciplinary approach by experts with the use of individualized medicine offering state-of-the-art sequencing technologies is essential for diagnosis in individuals with atypical forms of rare diseases.

## Data Availability

The datasets generated and/or analyzed during the current study are not publicly available due to protect individual’s privacy but are available from the corresponding author on reasonable request.

## Abbreviations

α-GAL: Alpha galactosidase A
Cr: Creatinine
DNA: Deoxyribonucleic acid
EM: Electron microscopy
ESRD: End stage renal disease
FD: Fabry disease
FSGS: Focal segmental glomerulosclerosis
Gb3: Globotriaosylceramide
IF: Immunofluorescence
LM: Light microscopy
Lyso-Gb3: Globotriaosylsphingosine
NPLRD: Nail-patella-like renal disease
NPS: Nail-patella syndrome
RNA: Ribonucleic acid
